# Effects of Quercetin Metabolites on Glucose‐Dependent Lipid Accumulation in 3T3‐L1 Adipocytes

**DOI:** 10.1002/mnfr.70070

**Published:** 2025-04-21

**Authors:** Marco Rendine, Samuele Venturi, Mirko Marino, Claudio Gardana, Peter Møller, Daniela Martini, Patrizia Riso, Cristian Del Bo

**Affiliations:** ^1^ Division of Human Nutrition, Department of Food, Environmental and Nutritional Sciences (DeFENS) Università degli Studi di Milano Milan Italy; ^2^ Department of Public Health University of Copenhagen Copenhagen Denmark

**Keywords:** adipocytes, AMPK, caloric restriction, (poly)phenols, quercetin

## Abstract

The aim of the study was to assess the effects of quercetin metabolites (QMs) on lipid accumulation in adipocytes under high‐glucose and physiological‐glucose concentrations and to elucidate the mechanisms involved. 3T3‐L1 mature adipocytes were exposed to a physiological glucose concentration, as a model of caloric restriction (CR), or high glucose (control), with and without QMs (quercetin‐3‐glucuronide [Q3G] and isorhamnetin [ISOR]). Cells were treated with Q3G (0.3 and 0.6 µmol/L) and ISOR (0.2 and 0.4 µmol/L) for 48 h. Lipid accumulation (Oil Red O staining) and Δ glucose level (HPLC) were assessed. Under high glucose, Q3G and ISOR reduced lipid accumulation (−10.8% and −10.4%; *p* < 0.01) and Δ glucose level (−13.6% and −14.2%; *p* < 0.05). Under CR, QMs increased Δ glucose level (+21.6% for Q3G and +21% for ISOR; *p* < 0.05). ISOR increased pAMPK levels under high glucose (+1.4‐fold; *p* < 0.05). Under CR, Q3G and ISOR increased pAMPK (+1.4‐ and +1.5‐fold; *p* < 0.05), while ISOR upregulated SIRT1 and PGC‐1α (+2.3‐ and +1.5‐fold; *p* < 0.05). Findings support, for the first time, the potential contribution of QMs, especially ISOR, in the regulation of lipid metabolism in vitro, possibly via AMPK activation. Further studies, including in vivo, are encouraged to strengthen evidence of the mechanisms observed.

## Graphical abstract

Quercetin metabolites (QMs), found in foods like fruits and vegetables, can influence how fat cells process fats and sugars. This study shows that QMs help reduce fat storage and sugar uptake in fat cells when sugar levels are high. Under conditions mimicking caloric restriction, they boost sugar use for energy. These effects are linked to activating specific proteins (AMPK, SIRT1, and PGC‐1α) that regulate metabolism. These findings suggest QMs may support healthier metabolism, paving the way for further research.

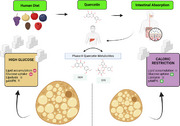

**Abbreviations**
ADP: adiponectin
CR: caloric restriction
DEXA: dexamethasone
DM: diabetes mellitus
IBMX: methylisobutylxanthine
ISOR: isorhamnetin
pAMPK: phosphorylated‐5' adenosine monophosphate‐activated protein kinase
PGC‐1α: peroxisome proliferator‐activated receptor gamma coactivator 1‐alpha
PPAR‐γ: peroxisome proliferator‐activated receptor‐γ
Q3G: quercetin‐3‐glucuronide
QMs: quercetin metabolites
SIRT‐1: sirtuin 1
UCP‐1: uncoupling protein 1

## 1 Introduction

Obesity has emerged as a significant public health challenge, associated with a spectrum of comorbidities including cardiovascular diseases (e.g., coronary heart disease, hypertension), various cancers, and non‐insulin‐dependent diabetes mellitus (DM), among others [1]. Obesity is associated with significant alterations in metabolic parameters, including dysregulation of glycemia and lipidemia, as well as impaired functionality of key organs involved in energy metabolism (e.g., liver, adipose tissue). Caloric restriction (CR), achieved by reducing caloric supply below the habitual intake in humans (or ad libitum feeding in animals) without deprivation of essential nutrients, helps reestablish normal metabolic parameters. However, maintaining weight loss achieved through CR remains a substantial challenge for many people [2, 3, 4]. Following weight and fat reduction, primarily induced by CR, a series of complex physiological adaptations occur, collectively creating a biological drive that may promote weight regain [5]. Indeed, after weight loss, a suppressed metabolic rate (also referred to as enhanced metabolic efficiency) has been observed in both humans [6, 7, 8, 9, 10, 11] and rodents [12, 13, 14] because of changes in neuro‐endocrine signals. These alterations include reduced sympathetic nervous system tone [15, 16, 17, 18], decreased thyroid hormone levels [19, 20, 21], and increased activity of the hypothalamic‐pituitary‐adrenal axis [22, 23, 24]. The adipose tissue plays a central role in the adaptations observed during weight loss. A key mechanism facilitating weight regain involves a reduction in leptin production by adipocytes, documented as a consequence of decreased lipid storage [21, 25]. Other molecular targets in adipocytes are widely known to regulate pivotal metabolic processes, including adipogenesis, lipogenesis, and thermogenesis. In this regard, the peroxisome proliferator‐activated receptor‐γ (PPAR‐γ) is a fatty acid sensor and master regulator of adipose tissue metabolism [26]. Another pivotal regulator of adipocytes’ functions is Sirtuin 1 (SIRT1), an NAD+‐dependent deacetylase that regulates primary cellular processes including lipogenesis and the inflammatory response [27]. Recent studies have highlighted the critical interplay between PPARγ and SIRT1 in regulating thermogenesis and mitochondria biogenesis [28], effects that are protective against obesity‐associated metabolic disorders [29]. Evidence suggests that CR and fat loss may promote mitochondria biogenesis [30, 31], yet the exact contribution of the PPARγ‐SIRT1 molecular network is not completely understood.

In this context, nutritional strategies that include the intake of foods rich in bioactive compounds may represent a synergistic approach for enhancing weight reduction and maintenance. Quercetin, a flavonoid widely present in the Mediterranean diet, has gained significant attention as a beneficial dietary factor [32]. Notably, quercetin primarily exists in its glucoside conjugate form, undergoing rapid metabolism in the liver to yield various metabolites, including glucuronide, methyl, and sulfate derivatives [33]. Consequently, a comprehensive exploration of the bioactivity of quercetin metabolites (QMs) within body tissues is essential for gaining a deeper understanding of its mechanism of action. Quercetin's effects have been investigated at the cellular level, especially at the level of adipose tissue, with diverse mechanisms proposed, indicating its potential to modulate key molecular pathways, such as those associated with adipogenesis and lipogenesis [34, 35], oxidative stress [36], inflammation [37, 38], and thermogenesis [39, 40]. However, most of these studies that hypothesized quercetin or its metabolites as anti‐obesogenic agents have primarily focused on pre‐adipocytes during their differentiation to adipocyte‐like cells [38, 39, 40, 41], a model that does not completely represent the condition of obesity. Moreover, there is no evidence regarding the potential effects of quercetin and its metabolites when a normal physiological condition (i.e., physiological glucose levels) is restored after an obesogenic condition (i.e., high glucose levels), mimicking a state of CR, which itself promotes lipid mobilization and oxidation.

This study aimed to evaluate, for the first time, the potential anti‐obesogenic activity of QMs and elucidate their mechanism of action under high and low caloric supply. In humans and animal models, excessive ingestion of food or calorie‐dense food items (e.g., Western‐type diet) results in high caloric intake. Therefore, CR typically entails a reduction of carbohydrates, lipids, and proteins. This situation cannot be entirely replicated in cell culture studies. Thus, we have regulated the calorie supply by the glucose concentration in the cell culture medium. High glucose refers to an unhealthy excessive calorie supply, whereas CR mimics the desired/normal condition. To this end, mature 3T3‐L1 adipocytes were used as an in vitro model of obesity, evaluating QMs under both high‐glucose and physiological‐glucose concentrations to simulate CR.

## 2 Experimental Section

### 2.1 Cells, Chemicals, and Reagents

Murine 3T3‐L1 cells from the European Collection of Authenticated Cell Cultures (ECACC, number 86052701‐1VL) were purchased from Sigma‐Aldrich (St. Louis, MO, USA). High glucose Dulbecco′s Modified Eagle′s Medium (high‐glucose DMEM, Cat. No. D0822‐500 mL), low‐glucose Dulbecco′s Modified Eagle′s Medium (low‐glucose DMEM, Cat. No. D6046‐500 mL), Oil Red O (Cat. No. O0625‐25 mg), bovine insulin (Cat. No. I0516‐5 mL), dexamethasone (DEXA, Cat. No. D4902‐100 mg), methylisobutylxanthine (IBMX, Cat. No. I5879‐250 mg), penicillin–streptomycin solution (Cat. No. P4333‐100 mL), trypsin‐EDTA (Cat. No. T4049‐100 mL), phosphate buffer saline (PBS; Cat. No. 806544‐500 mL), Trypan Blue (Cat. No. T8154‐100 mL), Triton X‐100, and formalin (Cat. No. R04586) were provided by Sigma‐Aldrich (St. Louis, MO, USA). Fetal Bovine Serum (FBS; Cat. No. A47668‐01) was purchased by Thermo Fisher Scientific (Waltham, MA, USA). ProteinSafe Protease Inhibitor Cocktail (100 ×) (Cat. No. DI111‐02) was provided by CliniSciences (Guidonia Montecelio, RM, Italy). Dimethyl sulfoxide (DMSO) was purchased from Merck (Darmstadt, Germany). Methanol and isopropanol were obtained from VWR International (Radnor, Pennsylvania, USA). Standards of glucose (Cat. No. G8270) and quercetin‐3‐glucuronide (Q3G, Cat. No. 16654‐10 mg) were purchased from Sigma‐Aldrich (St. Louis, MO, USA), while standard of isorhamnetin (ISOR, Cat. No.1120 S) was provided by Extrasynthese (Genay, France). Glycerol Assay Kit (Cat. No. MAK117) was purchased from Sigma‐Aldrich (St. Louis, MO, USA). ELISA kits used to quantify the protein levels of adiponectin (ADP, Cat. No. MBS2702138‐96), phosphorylated‐5' adenosine monophosphate‐activated protein kinase (pAMPK, Cat. No. MBS2505028‐96), PPAR‐γ (Cat. No. MBS2021822‐96), sirtuin‐1 (SIRT‐1, Cat. No. MBS451811‐96), peroxisome proliferator‐activated receptor gamma coactivator 1‐alpha (PGC‐1α, Cat. No. MBS452675‐96) and uncoupling protein 1 (UCP‐1, Cat. No. MBS933377‐96) were purchased from MyBioSource, Inc. (San Diego, CA, USA).

### 2.2 Preparation of Stock Solutions

Stock solutions of QMs were prepared by dissolving compounds in methanol at a concentration of 1 mg/mL and storing them at −20°C until use. We used methanol because it is widely recognized as the ideal solvent for polyphenol extraction from food matrices. Moreover, methanol has been used in previous studies on polyphenol extracts in cell models [42, 43]. The stock stain at Oil Red O (0.5 % w/v) was prepared in isopropanol and stored at 4°C. Working solutions of experimental concentrations for each compound were freshly prepared before each experiment.

### 2.3 Cell Culture and Differentiation of 3T3‐L1 Cells

Murine 3T3‐L1 pre‐adipocytes were cultured in high‐glucose DMEM (4.5 g/L glucose + pyruvate) with 10% FBS supplemented with 0.5% antibiotics (penicillin/streptomycin). Culture medium was changed every 2–3 days. 3T3‐L1 cells were differentiated into mature adipocytes through a standard differentiation protocol [44] with slight modifications. In this work, differentiation refers to the process by which 3T3‐L1 fibroblasts acquire lipid‐storing capacity through key transcription factors such as PPARγ and C/EBPα, leading to the formation of adipocyte‐like cells, a process known as adipogenesis [45]. Another type of process is the browning process, which involves the transdifferentiation of mature adipocytes into beige adipocytes through the activation of thermogenic pathways, such as PGC‐1α and UCP1, in response to environmental cues like cold exposure [44]. Briefly, after seeding in experimental plates, cells were grown until reaching confluence. Then, 2 days post confluence (designed as Day 0), cells were exposed to an induction medium consisting of DMEM (with 10% FBS and 0.5% antibiotics) supplemented with IBMX (500 µM), DEXA (1 µM), and insulin (1 µg/mL) for 48 h. On Day 3, the induction medium was replaced with insulin medium (IM), consisting of DMEM with 10% FBS, 0.5% antibiotics, and 1 µg/mL insulin. Differentiating cells were cultured in fresh IM after 48 h. On Day 7, 3T3‐L1 adipocytes showed a differentiated phenotype with massive accumulation of fat droplets and were used for experiments. Mature adipocytes were treated for 24 or 48 h with QMs at physiological (i.e., Q3G 0.3 µM, ISOR 0.2 µM) or super‐physiological concentrations (i.e., Q3G 0.6 µM, ISOR 0.4 µM) under both CR and standard conditions (control, CTR). In vitro CR was obtained by culturing adipocytes in a normal physiological glucose concentration (DMEM, glucose 1 g/L), while the high‐glucose condition (DMEM, glucose 4.5 g/L) was used as the control group. Treatments were administered in serum‐free DMEM (high‐ or normal‐glucose) with 0.5% antibiotics. For all experiments, cells were used between passages 2 and 10 and cultured at 37°C and 5% CO_2_.

### 2.4 Cell Viability and Cytotoxicity Assays

The potential cell death and cytotoxicity of QMs has been evaluated with the Trypan Blue exclusion assay and MTT [3‐(4,5‐dimethylthiazol‐2‐yl)‐2,5‐diphenyltetrazolium bromide] assays. The Trypan Blue assay detects dead cells, whereas the MTT assay detects both dead and cytotoxic cells. 3T3‐L1 preadipocytes were seeded in 24‐well plates at a density of 2 × 10^4^ cells/well. Cell cultures with 80% confluence were treated with QMs at different concentrations (0.2–0.6 µM) for 48 h. Cell culture medium was used as a negative control(s) (CTR and CR), and 0.1% Triton X‐100 (TX‐100) as a positive control. Subsequently, cells were detached with trypsin/EDTA (0.05%), resuspended, and analyzed with a Trypan Blue exclusion assay using a TC20TM automated cell counter and dedicated dual‐chamber cell counting slides (BIORAD, Segrate, Milan, Italy). Three independent experiments were conducted, with each experimental condition assessed in duplicate within each experiment. Additionally, the cytotoxicity was evaluated through an MTT assay. Cells were seeded in 96‐well plates (5 × 10^3^ cells/well). After 48 h, cells were treated with the QMs at different concentrations (0.2–0.6 µM), CTR, CR, and TX‐100. The MTT solution, prepared in a cell culture complete medium (1 mg/mL), was filter‐sterilized (0.2 µm diameter), and 100 µL of the MTT solution was dispensed into each well of the cell culture plate. Cells incubated with the MTT solution were maintained at 37°C for 4 h under light‐free conditions. Following incubation, the MTT solution was removed, and 200 µL of DMSO was added to each well to dissolve the formazan crystals. The plate was then placed on a diametral shaker in darkness for 20 min at room temperature. Absorbance readings were obtained at 540 nm using a plate reader (mod. F200, TECAN, Milan, Italy) [47]. Each experimental condition was evaluated in four replicates across three independent experiments.

### 2.5 Cellular Lipid Accumulation

The intracellular lipid content has been evaluated by the semi‐quantitative Oil Red O staining assay. 3T3‐L1 preadipocytes were seeded in 24‐well plates at a density of 2 × 10^4^ cells/well and differentiated to adipocytes. Mature adipocytes were treated with QMs at different concentrations (0.2–0.6 µM) within low‐ and high‐glucose serum‐free DMEM for 48 h. The highest percentage of methanol (0.1%) occurred in the treatment with the highest concentration of ISOR (0.4 µM). Therefore, the same quantity of methanol was added as a vehicle to negative control(s) (CTR and CR). After treatments (on Day 9), culture medium was collected for glycerol and glucose quantification, then adipocytes were washed with 0.5 mL of PBS and then incubated with 0.5 mL of formalin (10%) for 30 min at room temperature (≈23°C). Subsequently, cells were washed twice with 0.5 mL of PBS, and 0.5 mL of 60% isopropanol was added for 5 min. Later, the liquid layer was discarded, and 0.5 mL of Oil Red O working solution 0.2% (prepared by diluting stock solution 2:3 in distilled water) was added to the cells. The plates were incubated in the dark (at room temperature for 1 h) to allow the staining of lipids with Oil Red O solution, then cells were washed 3 times with distilled water to remove the residuals. Finally, 0.5 mL of 99% isopropanol was added to cells to extract the dye, and the absorbance of the resulting solutions was read at 550 nm using a microplate reader (mod. F200 Infinite, TECAN, Milan, Italy). Three independent experiments were performed in which each condition was tested in duplicates.

### 2.6 Lipolytic Activity

To evaluate the lipolytic activity of adipocytes, the concentration of glycerol released into the cell medium was measured after 48 h of treatments. High glucose exposure promotes lipogenesis in 3T3‐L1 adipocytes, which entails a de novo production of glycerol‐3‐phosphate [48]. Thus, lipolytic activity refers to the effects of QMs within the group of either high or low glucose content in cell culture medium. The same treatment phases of the lipid accumulation assay described above were followed. Subsequently, cell medium was collected and centrifuged at 250 × *g* for 10 min at 4°C and stored at −80°C until analysis. Glycerol levels were quantified using a colorimetric kit (Sigma, MAK117) according to the manufacturer's instructions. Each condition was evaluated in duplicate in three separate experiments.

### 2.7 Delta Glucose Level Analysis

Glucose concentration was determined in cell media through a high‐performance liquid chromatography (HPLC) system as previously described by Gardana et al. [49]. Briefly, the chromatographic system consisted of an Alliance model 2695 (Waters) equipped with a model 2420 (Waters) evaporative light scattering detector (ELSD). A 5 mm Prevail Amino column (250 × 4.6 mm, Alltech) was used for the separation in isocratic mode at a flow rate of 1.5 mL/min, and the eluent was acetonitrile:water (80:20, v/v). The column and the sample were maintained at 35 and 20°C, respectively. The ELSD conditions were the following: gain 100, pressure 40 psi, and drift tube temperature 40°C. All data were acquired by Empower 2 software (Waters). Calibration curves were obtained from glucose stock solutions prepared by dissolving 200 mg of standard powder in 100 mL of water. The working solutions in water:acetonitrile (1:1, v/v) were prepared in the range of 0.2–1 mg/mL. Fifty milliliters were injected in the LC system.

The same treatment phases of lipid accumulation assay described above were followed. Cell media were collected after each experiment, centrifuged at 250 × *g* for 10 min at 4°C, and stored at −80°C until analysis. Delta (Δ) glucose levels were determined by measuring the change in glucose concentration between spent medium after treatments and the fresh medium samples. Fresh medium was subjected to the same processes as the experimental samples (i.e., incubated for 48 h, centrifuged, and stored at −80°C until analysis). Three independent experiments were conducted, with each condition tested in duplicate.

### 2.8 Protein Extraction and Evaluation of Thermogenic and Lipogenic Markers

To evaluate markers of thermogenesis and lipogenesis, cells were seeded in a 6‐well plate at a density of 8 × 10^4^ cells/well. Cells were differentiated into mature adipocytes for 7 days. On Day 7, mature adipocytes were treated for 24 h with or without QMs (i.e., Q3G 0.6 µM, ISOR 0.4 µM) within standard conditions (high glucose) and CR (low glucose). The highest quantity of methanol (0.1%) was added as a vehicle to negative control(s) (CTR and CR). Cell medium was collected to evaluate ADP levels, while cell lysate was collected to measure protein levels of pAMPK, PPAR‐γ, SIRT‐1, PGC‐1α, and UCP‐1 using enzyme‐linked immunosorbent assay (ELISA). Protein extraction was completed in accordance with the kit's protocol. Briefly, cell medium was collected, centrifuged at 300 × *g* for 10 min at 4°C, and stored at −80°C until analysis. Adipocytes were detached by trypsin/EDTA (0.05%). Subsequently, cells were washed with cold PBS and centrifuged at 300 × *g* for 5 min at 4°C three times. Then, cells were homogenized in a cold PBS buffer with 1% Protease Inhibitor Cocktail and 1% EDTA. Cell lysis was obtained by four cycles of ultrasonication for 90 s (in cold water) followed by vortexing for 15 s. Next, the cell extract was centrifuged at 2000 × *g* for 15 min at 4°C. The resulting supernatant containing intracellular protein was collected and immediately stored at −80°C until analysis. Three independent experiments were performed in which each condition was tested in duplicate.

### 2.9 Statistical Analysis

Data were initially analyzed to identify outliers by the ROUT method and for their normal distribution using Shapiro–Wilk test. Then, one‐way analysis of variance (ANOVA) was used to evaluate the effects of QMs within high‐ and low‐glucose on lipid accumulation, lipolytic activity, and Δ glucose level. A two‐way ANOVA has been performed for the analysis of thermogenic and lipogenic markers in mature 3T3‐L1 adipocytes. Post‐hoc analysis of differences between treatments has been conducted by the Fisher's least significant difference (LSD) test with a level of statistical significance set at *p* ˂ 0.05. Statistical analysis was conducted using GraphPad Prism 8.4.2 (GraphPad Software Inc., San Diego, CA).

## 3 Results

### 3.1 Effects of QMs on 3T3‐L1 Cell Viability and Cytotoxicity

Table 1 reports the results on cell viability evaluated after 48 h of treatment with the highest concentrations of QMs. The viability and cytotoxicity have been evaluated by Trypan Blue exclusion assay and by MTT assay. Data obtained from both assays agreed. It is worth mentioning that the positive control (TX‐100) significantly reduced cell viability values. On the other hand, when Q3G and ISOR were used as treatment, both within low‐ and high‐glucose, cell viability remained above 85%.

**TABLE 1:** Data are mean (%) with respect to control (high glucose) ± standard deviation (SD) of three independent experiments, with each condition performed in triplicate.

Treatment	Cell viability/cytotoxicity (% with respect to control) MTT	Cell viability (% with respect to control) Trypan Blue exclusion
Untreated adipocytes		
High glucose (Control) Low glucose (CR)	100.0 ± 11.4 86.4 ± 12.5	100.0 ± 2.5 104.6 ± 1.8
Quercetin‐3‐glucuronide (0.6 µmol/L) High glucose Low glucose	92.3 ± 24.0 86.6 ± 27.3	98.2 ± 6.2 103.0 ± 2.1
Isorhamnetin (0.4 µmol/L) High glucose Low glucose	93.7 ± 18.1 114.8 ± 29.7	102.5 ± 2.6 103.0 ± 2.1
TX‐100	***2.1 ± 1.4	***14 ± 10.6

*p < 0.05, **p < 0.01, ***p < 0.001.

### 3.2 Effect of Q3g and ISOR on Lipid Accumulation in Mature Adipocytes

**FIGURE 1:**
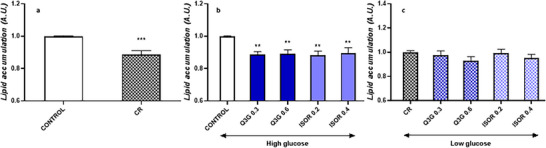
Effect on adipocytes’ lipid accumulation determined by Oil Red O staining assay. (a) Effects on adipocytes’ lipid accumulation after 48 h incubation under two different concentrations of glucose (control, 4.5 g/L vs. caloric restriction [CR, 1 g/L]). (b) Effects on adipocytes’ lipid accumulation after 48 h incubation with quercetin metabolites under high‐glucose condition. (c) Effects on adipocytes’ lipid accumulation after 48 h incubation with quercetin metabolites under low‐glucose condition. Data are presented as mean ± standard error of the mean (SEM) of three independent experiments, with each condition performed in duplicate. *p < 0.05, **p < 0.01, ***p < 0.001, ****p < 0.0001.

Figure 1a presents the results related to fat deposition following the incubation of mature adipocytes (on Day 7 post‐differentiation) with two different concentrations of glucose (high‐ and low‐glucose, 4.5 g/L and 1 g/L, respectively) for 48 h. Adipocytes cultured in low glucose concentration (i.e., CR condition) significantly reduced their fat deposits (−10.6%; *p* < 0.001) compared to the control (high‐glucose culture medium). Subsequently, Q3G and ISOR were administered to mature adipocytes with both concentrations of glucose, and their effect on fat storage was assessed after 48 h. Under high‐glucose condition, data indicated that the administration of physiological doses of both Q3G and ISOR reduced lipid accumulation (Figure 1b). Q3G resulted in a significant lipid reduction of −11.4% and −10.8%, *p* < 0.01 (when tested at 0.3 and 0.6 µmol/L, respectively) with respect to untreated control. ISOR reduced lipids by −11.8% and −10.4%, *p* < 0.01 (at 0.2 and 0.4 µmol/L, respectively) with respect to the untreated control. Conversely, when QMs were tested under low‐glucose condition, no significant reduction in lipid content of mature adipocytes was observed compared to the glucose‐restricted control (CR) (Figure 1c).

### 3.3 Effect of Q3g and ISOR on Lipolytic Activity in Mature Adipocytes

**FIGURE 2:**
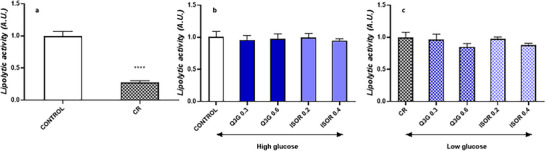
Effect on adipocytes’ lipolytic activity for 48 h, determined by glycerol quantification in cell culture medium. (a) Effects on adipocytes’ lipolytic activity after 48 h incubation under two different concentrations of glucose (control, 4.5 g/L vs. caloric restriction [CR, 1 g/L]). (b) Effects on adipocytes’ lipolytic activity after incubation with quercetin metabolites under high‐glucose condition. (c) Effects on adipocytes’ lipolytic activity after incubation with quercetin metabolites under low‐glucose condition. Data are reported as mean ± standard error of the mean (SEM) of three independent experiments, with each condition performed in duplicate. *p < 0.05, **p < 0.01, ***p < 0.001, ****p < 0.0001.

Figure 2 shows the levels of glycerol released by 3T3‐L1 adipocytes after treatments with QMs administered within high‐ and low‐glucose medium. Glycerol released in cell culture medium was quantified spectrophotometrically as a marker of lipolytic activity. Levels of glycerol in cell culture medium were significantly reduced in the CR group compared to the control (high‐glucose culture medium) (Figure 2a). Neither Q3G nor ISOR modulated lipolysis in mature adipocytes (Figure 2b,c).

### 3.4 Effect of Q3G and ISOR on Δ Glucose Level in Cell Supernatants

**FIGURE 3:**
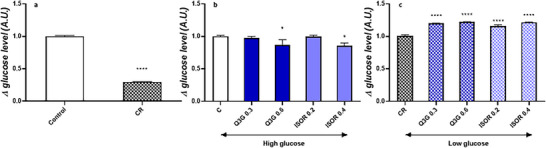
Effect on changes in glucose concentration (Δ glucose level) in cell supernatants after 48 h, determined by HPLC. The Δ glucose level was measured by determining the difference in glucose concentration before and after the experiments. (a) Effects on Δ glucose level after 48 h incubation of adipocytes under two different concentrations of glucose (control, 4.5 g/L vs. caloric restriction [CR, 1 g/L]). (b) Effects on Δ glucose level after 48 h incubation of adipocytes with quercetin metabolites under high‐glucose condition. (c) Effects on Δ glucose level after 48 h incubation of adipocytes with quercetin metabolites under low‐glucose condition. Data are presented as mean ± standard error of the mean (SEM) of three independent experiments, with each condition performed in duplicate. *p < 0.05, **p < 0.01, ***p < 0.001, ****p < 0.0001.

Since glucose is the primary substrate utilized by cultured cells, which in adipocytes is used to synthesize free fatty acids and increase lipid droplet formation, the effects of Q3G and ISOR on insulin‐independent glucose consumption (Δ glucose level) in mature adipocytes under varying glucose concentrations were tested, and results are reported in Figure 3. Figure 3a reports the results on Δ glucose level in cell supernatants following the incubation of mature adipocytes (on Day 7 post‐differentiation) with two different concentrations of glucose (high‐ and low‐glucose, 4.5 g/L and 1 g/L, respectively) for 48 h. The Δ glucose level significantly decreased (*p* < 0.0001) in adipocytes cultured in low‐glucose medium (CR condition) compared to the control (high‐glucose culture medium). QMs modulated the Δ glucose level in cell supernatants under both high‐ and low‐glucose conditions. Particularly, when administered within high‐glucose culture medium, Q3G at 0.6 µmol/L and ISOR at 0.4 µmol/L significantly reduced the Δ glucose level (*p* < 0.05) to an extent of −13.6% and −14.2%, respectively, compared to the untreated control (Figure 3b). No significant effect was observed with the lowest concentration tested of QMs within high‐glucose cell culture medium (Figure 3b). On the contrary, under CR, both experimental concentrations of Q3G and ISOR produced a significant increase in the Δ glucose level (*p* < 0.0001) compared to the respective low‐glucose control (Figure 3c). In particular, Q3G increased the Δ glucose level by 19% and 21.6% at 0.3 and 0.6 µmol/L, respectively, while ISOR enhanced the Δ glucose level by 14.8% and 21% at 0.2 and 0.4 µmol/L, respectively.

### 3.5 Effect of Q3G and ISOR on Thermogenic and Lipogenic Markers in Mature Adipocytes

**FIGURE 4:**
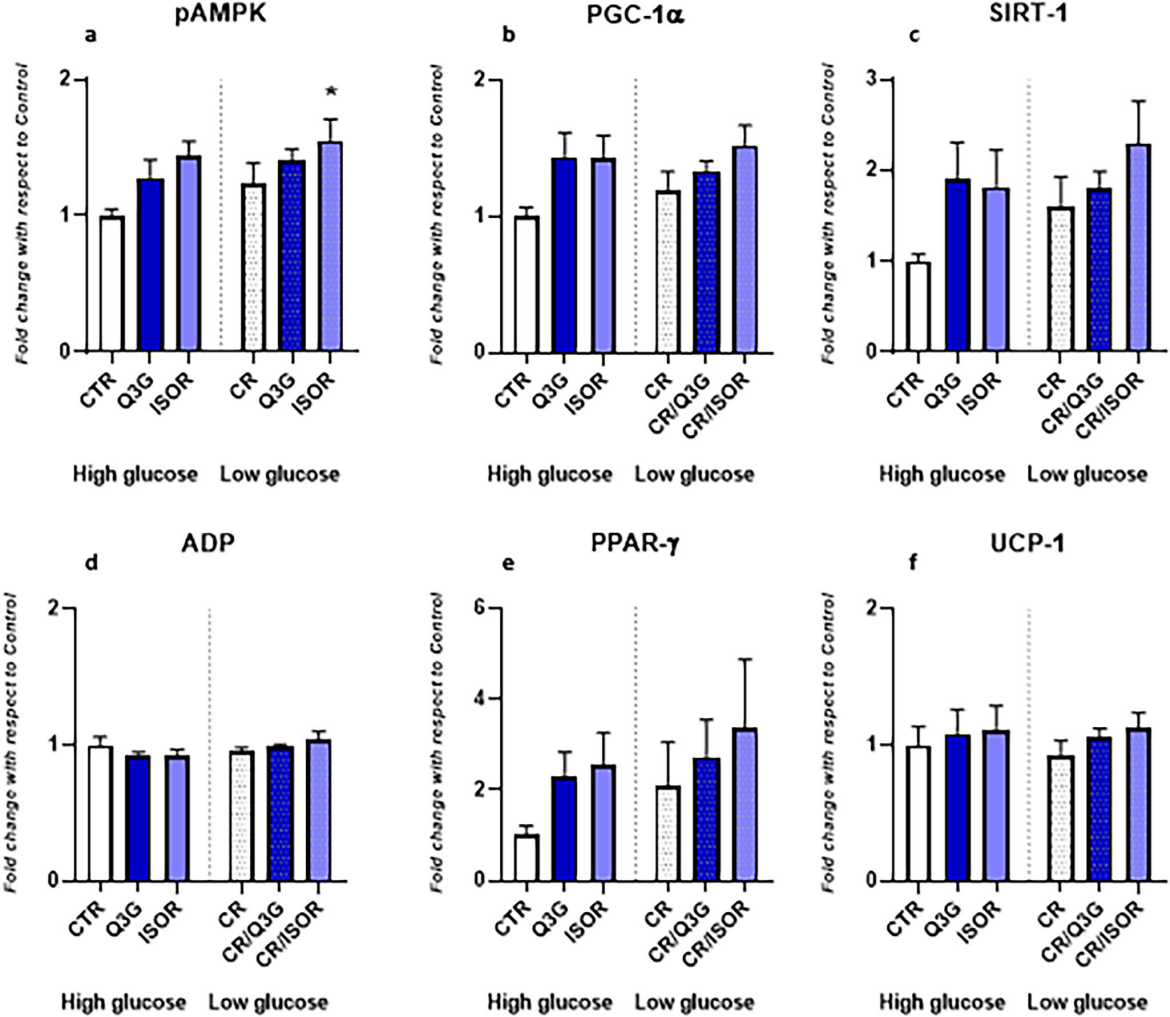
Effect on adipocytes’ markers related to lipogenesis and thermogenesis. (a) Effects on phosphorylated‐ 5' AMP‐activated protein kinase (pAMPK) protein levels after incubation with quercetin metabolites under high‐glucose and low‐glucose conditions. (b) Effects on peroxisome proliferator‐activated receptor‐gamma coactivator‐1alpha (PGC‐1α) protein levels after incubation with quercetin metabolites under high‐glucose and low‐glucose conditions. (c) Effects on sirtuin‐1 (SIRT‐1) protein levels after incubation with quercetin metabolites under high‐glucose and low‐glucose conditions. (d) Effects on adiponectin (ADP) protein levels after incubation with quercetin metabolites under high‐glucose and low‐glucose conditions. (e) Effects on peroxisome proliferator‐activated receptor‐gamma (PPAR‐γ) protein levels after incubation with quercetin metabolites under high‐glucose and low‐glucose conditions. (f) Effects on uncoupling protein‐1 (UCP‐1) protein levels after incubation with quercetin metabolites under high‐glucose and low‐glucose conditions. Data are presented as mean ± standard error of the mean (SEM) of three independent experiments, with each condition performed in duplicate. *p < 0.05, **p < 0.01, ***p < 0.001, ****p < 0.0001.

After treating mature adipocytes with QMs under high‐ and low‐glucose conditions, protein levels of markers related to thermogenesis and lipogenesis were evaluated. The results indicated that glucose concentration per se did not affect the expression of proteins analyzed. Moreover, there was no interaction effect between QM exposure and glucose condition. Regarding the effect of treatments, under high‐glucose condition, QMs did not produce statistically significant effects on the levels of proteins investigated, although there were weak (statistically non‐significant) increases of pAMPK, PGC‐1α, and SIRT‐1. In contrast, under low‐glucose conditions, the treatment with ISOR significantly increased pAMPK (*p* = 0.035) (Figure 4a). Additionally, although not statistically significant, after the treatment with ISOR, a minor upregulation of PGC‐1α (Figure 4b) and SIRT‐1 (Figure 4c) was observed under low glucose condition (*p* = 0.097 and *p* = 0.102, respectively). Finally, neither ISOR nor Q3G were able to affect protein levels of ADP, PPAR‐γ, and UCP‐1 in both glucose conditions (Figure 4d–f).

## 4 Discussion

The present study aimed to investigate the effects of physiological concentrations of two main phase II‐derived QMs, specifically Q3G and ISOR [33], on the capacity to counteract lipid accumulation in mature 3T3‐L1 adipocytes under different glucose conditions. Although QMs may be present in small amounts in food, the glucuronidated and methylated quercetin derivatives found in human plasma are primarily produced through intestinal and hepatic metabolism [50]. The concentrations of Q3G and ISOR were selected in accordance with data from the literature that have shown an increase in plasma levels following the consumption of quercetin‐rich foods (e.g., onion, apples). For example, Mullen and colleagues [51] showed that Q3G appeared in plasma at the concentration of ∼ 350 nM after the consumption of 270 g of dried fried onion. Day et al. [52] reported a plasma concentration of 260 nM of Q3C following the consumption of 200 g of fried onions. Day et al. [52] and Hubbard et al. 2006 [53] observed plasma levels of ISOR in the range of 100–200 nM after the consumption of different onion‐food products (e.g., fried onion, onion soup).

We have shown that Q3G and ISOR promote a reduction in lipid accumulation, measured by Oil Red O staining, in mature adipocytes under high‐glucose conditions. Several studies have evaluated the role of quercetin, QMs, or quercetin‐rich extracts on lipid accumulation in 3T3‐L1 cells during their differentiation to mature adipocytes. For instance, Lee et al. [41] found a reduction of lipid accumulated in 3T3‐L1 cells following the administration of an onion peel extract, containing quercetin and isoquercetin as its main flavonoids. Similar findings were documented in another study in which the effect of quercetin (10–80 µg/mL) on lipid accumulation was tested in 3T3‐L1 adipocytes [54]. Nonetheless, these and other studies [38, 39, 41, 55–58] have demonstrated an effect of extracts and single compounds on 3T3‐L1 preadipocytes. It is crucial to note that genetic plasticity changes throughout the differentiation process [59, 60, 61], thus testing bioactive compounds on adipocyte‐like cells at the end of the differentiation process may yield different results, highlighting the importance of considering the stage of adipocyte development when interpreting the outcomes. Additionally, during differentiation, these compounds are tested alongside other agents used to induce differentiation (i.e., IBMX, DEXA, and insulin), which may lead to potential synergistic effects among the compounds, making it difficult to discriminate the specific effects of each compound. Regarding studies focusing on the effect of quercetin on lipid accumulation in mature 3T3‐L1 adipocytes, the results are in line with our observations. For instance, Aranaz et al. [60] evidenced a decrease in lipid accumulation after treatment for 8 days with quercetin (at the concentration of 100 µM). In another work, quercetin, and its metabolites (i.e., ISOR, tamarixetin, quercetin‐3‐O‐glucuronide, quercetin‐3‐O‐sulfate, and quercetin‐4‐O‐sulfate) have been proved to reduce intracellular lipids after the treatment of mature adipocytes for 24 h [34]. Moreover, another study showed the capacity of Q3G (25–100 µM) in reducing lipid accumulation in hypertrophic adipocytes cultured in high‐glucose concentration (25 mM, corresponding to 4.5 g/L) [35]. However, in all these studies the effect on lipid depots was mainly evidenced at pharmacological or supraphysiological concentrations (10‐100 µM), thus not representative of a real‐life condition or translatable/applicable in a nutritional context.

In our experiments, we also evaluated the effect of QMs under low‐glucose conditions mimicking CR. In this case, the reduction in lipid content was not significant, suggesting that QMs do not exert a synergistic effect at low glucose condition. In this context, a study conducted by Eldaim et al. [63] evidenced the glucose‐dependent effect on lipid accumulation exerted by retinoic acid, suggesting the potential differential effects of bioactive compounds depending on glucose availability. In the presence of glucose at the concentration of 5 mM, the addition of retinoic acid produced less lipid accumulation in adipocytes compared to the cells cultured in the same glucose condition but without the retinoic acid. On the contrary, when cells were cultured in the presence of a higher glucose concentration (25 mM), the addition of retinoic acid led to an increased lipid accumulation.

The results observed in our experiments using the 3T3‐L1 adipocytes agree with the anti‐obesogenic effect of quercetin evidenced in some animal studies [64, 65]. Jiang and colleagues [64] treated adult male ICR mice with quercetin and its glycosides in the context of a standard diet and a high‐fat diet. The authors reported that administration of quercetin glycosides (0.1% of the diet) for 13 weeks decreased body weight gain and fat accumulation in the mesenteric adipose tissue of mice on high‐fat diets. Also, another study evaluated the effectiveness of a quercetin‐rich supplement (185 mg per kg of body weight) in high‐fat diet‐induced obese rats [65]. Results regarding adipose tissue histology showed that the quercetin‐supplemented group had reduced lipid accumulation and smaller adipocyte size.

In the current work, we also investigated the effects of QMs on glycerol release as an indicator of lipolysis in mature adipocytes. While Oil Red O staining provides a direct measure of intracellular lipid accumulation (mainly neutral triglycerides), glycerol release reflects the breakdown of stored triglycerides, offering insight into the dynamic regulation of lipid metabolism. These two methods are complementary but distinct, as lipid accumulation assesses net storage, whereas glycerol release indicates mobilization of triglycerides through lipolysis. In our study, we did not observe changes in lipolytic activity following Q3G and ISOR administration in the high‐glucose medium. These results agree with data obtained in another experiment in which QMs (i.e., quercetin‐3‐O‐sulfate and quercetin‐4‐O‐sulfate at 10 µM) were unable to affect the lipolysis in 3T3‐L1 adipocytes in the low‐glucose condition, suggesting that the observed lipid reduction is likely not mediated through direct lipolytic pathways but by other cellular mechanisms [34].

Glucose is the primary energetic substrate in cell culture. Mature adipocytes internalize glucose via insulin‐dependent and insulin‐independent transporters (i.e., GLUT4 and GLUT1, respectively). Glucose is rapidly metabolized through glycolysis to produce acetyl‐CoA, which in turn can enter the Krebs cycle or be channeled to biosynthesis of free fatty acids that can be stored as triglycerides in lipid vacuoles. Several studies reported the capacity of (poly)phenols to modulate the glucose uptake and metabolism in adipose tissue, thus regulating lipogenesis and lipid deposition [66, 67, 68, 69, 70]. In the present study, we evaluated whether QMs may affect the insulin‐independent glucose consumption (Δ glucose level) in adipocytes. However, the change in glucose concentration in the culture media, measured by the glucose delta method, reflects both cellular glucose uptake and metabolism. Despite this approach having been used previously [71], other more accurate methodologies, such as fluorescently tagged glucose analogs, could be used for assessing glucose uptake in adipocytes and other cell types [66, 68].

Our findings reveal that both Q3G and ISOR can effectively reduce the Δ glucose level in cell supernatants under high‐glucose condition. In this context, Lim et al. [67] investigated the effect of quercetin glycosides (e.g., rutin and quercetin‐3‐O‐β‐D‐glucoside) on glucose uptake in mature adipocytes, showing that compounds increased the insulin‐stimulated glucose uptake without significant effect on the insulin‐independent glucose uptake. While our study utilized the same cell model as the study by Lim et al. [67], a notable distinction lies in the compounds tested. In fact, the previous study assessed flavonoid glycosides, which are less likely to reach peripheral cells like adipocytes in vivo, while the quercetin‐derived metabolites in our experiments are also found in plasma, potentially exerting biological activity on adipose tissue cells. This difference in compound selection could account for the divergent findings observed on insulin‐independent glucose uptake.

Additionally, we assessed the effect of QMs on Δ glucose level under CR. Interestingly, our results revealed an increase in Δ glucose level under low‐glucose conditions, suggesting greater glucose internalization by adipocytes over 48 hours. These findings indicate that the effect induced by QMs was dependent on glucose concentration. These observations suggest a complex interplay between QMs, glucose availability, and adipocyte glucose metabolism. In this context, normal glucose levels per se are associated with adequate insulin sensitivity and glucose metabolism [72, 73, 74]. Additionally, it has been proposed that quercetin can reduce insulin resistance as well as improve glucose tolerance [75, 76], particularly in adipose tissue [37]. This mechanism has been further proved by experiments showing that quercetin can increase insulin‐dependent glucose uptake through the expression of GLUT4 in 3T3‐L1 cells [37] and other cell models [77]. Under high glucose conditions, QMs could act as glucose transporters’ competitors, a mechanism previously described [78].

In our study, the evaluation of thermogenic and lipogenic markers revealed distinct effects of QMs. Among these, AMPK is a cellular energy sensor and metabolic regulator, overseeing the balance of lipid and glucose metabolisms [79, 80], and its activation through phosphorylation in peripheral tissues (e.g., adipose tissue) mitigates obesity and related metabolic disorders [81, 82, 83]. Our findings demonstrate that ISOR activates AMPK in the context of CR (low‐glucose conditions), highlighting its potential involvement in energy homeostasis. AMPK activation leads to the inhibition of downstream targets involved in lipogenesis, including ACC1 and ACC2, which mediate the conversion of acetyl‐CoA to malonyl‐CoA [84], regulating the synthesis of fatty acids. However, the role of pAMPK in controlling glucose uptake in adipose tissue is still controversial. The main evidence, derived from studies in rats or in 3T3‐L1 adipocytes, suggests that activated AMPK may result in an increase in insulin‐stimulated glucose uptake as well as an enhancement of basal glucose uptake via GLUT4 and GLUT1, respectively [85, 86, 87]. Our data also suggests that ISOR modulates changes in glucose concentration in a glucose‐dependent manner. While AMPK activation is a plausible mechanism, further studies are needed to confirm its direct involvement. The implication of AMPK could be explained by its glucose‐sensing mechanisms. It is widely known that AMPK is a glucose sensor, activated by glucose deprivation [87]. In our study, the condition of CR slightly increased pAMPK levels, although not to the extent seen in studies on glucose starvation in glucose‐free cell culture medium. This could, at least in part, explain the modest activation of AMPK in our study under CR conditions, but not under high‐glucose conditions. Thus, it could be postulated that the increased Δ glucose level under CR induced by ISOR is a consequence of the synergistic effect of the compound and glucose restriction in activating AMPK. Overall, the increase in pAMPK observed in experiments under CR conditions suggests a potential role for AMPK modulation, although additional experiments are required to establish causality. However, our findings seem to be aligned with data reported by other authors. For instance, Wang et al. showed that the administration of a Q3G‐rich extract (128.2 µg/mg) induces the expression of mediators that promote the development of brown‐like adipocytes from mesenchymal stem cells, including SIRT‐1, PGC‐1α, UCP‐1, and cell death activator CIDE‐A [88]. Moreover, treatment with Q3G triggered AMPK phosphorylation. However, the pretreatment with an AMPK inhibitor reversed these changes, suggesting pAMPK as the primary mediator for the development of brown‐like adipocyte features [88]. In our study, we have also investigated molecular targets of pAMPK, including SIRT‐1, PGC‐1α, and UCP‐1 [89, 90], as well as molecules strictly related to the induction of AMPK, such as ADP and PPAR‐γ [91, 92]. Treatments with QMs did not affect the protein levels of ADP, PPAR‐γ, and UCP‐1 in mature adipocytes. Although we did not observe statistical significance, when tested in the model of CR, ISOR slightly upregulated SIRT‐1 and PGC‐1α. These results link well with the role of AMPK in the regulation of mitochondrial energetics and oxygen consumption in 3T3‐L1 adipocytes, as previously reported [93]. In fact, as mentioned above, there is a complex bidirectional interplay between AMPK and SIRT‐1. SIRT‐1 can promote AMPK activation by deacetylation of the upstream kinase LKB1, which in turn interacts with and activates AMPK in the cytoplasm [94]. AMPK, in turn, can enhance SIRT1‐dependent pathways through various mechanisms, such as disrupting the interaction between SIRT‐1 and its inhibitor (i.e., DBC1) [95, 96]. On the other hand, SIRT‐1 has been reported to increase PGC‐1α activity, improving mitochondrial mass and respiratory capacity [28]. Overall, it can be hypothesized that the activation of the pAMPK‐SIRT1‐PGC‐1α pathway under CR is responsible for the enhancement of glucose uptake without increasing lipid droplets in adipocytes.

The results obtained in this study contribute to the understanding of the potential roles of Q3G and ISOR in adipocyte function and activity. Our findings suggest that QMs may have similar effects, particularly in their glucose‐dependent biological impact on adipocytes. Our results indicate that under high glucose availability, these compounds reduce lipid accumulation and Δ glucose level. However, under CR conditions, Δ glucose level in cell supernatants increases without a significant change in lipid depots, suggesting enhanced energy dissipation. A putative mechanism of action could be associated with AMPK activation, which in previous studies has been linked to the regulation of SIRT‐1 and PGC‐1α, resulting in a high mitochondrial density and glucose oxidation. However, since our data do not provide direct evidence of this pathway, further studies are necessary to confirm the involvement of AMPK, elucidate the molecular pathways activated by Q3G and ISOR, and identify which QMs are responsible for the observed biological activity of quercetin‐rich food items, as both active and inactive forms may exist. Additionally, further molecular studies investigating the selective effects of QMs under varying glucose conditions are needed to better clarify the mechanisms of action of these compounds in relation to different levels of cell nutrient availability.

## 5 Concluding Remarks

In conclusion, this comprehensive study sheds light on the multifaceted effects of Q3G and ISOR on 3T3‐L1 adipocytes. The findings provide valuable insights into the potential role of QMs in managing lipid content in adipose tissue, notably in cells exposed to high glucose levels. Additionally, the glucose‐dependent effect of QMs highlighted an important interplay between glucose concentration and biological effects, suggesting that the activation of AMP kinase may result in different outcomes based on glucose availability. Further investigations are warranted to validate the underlying molecular mechanisms and extend these promising results in vivo studies. Given the complexity of whole‐body metabolism and inter‐organ crosstalk, in vivo studies are fundamental to confirm the efficacy and physiological relevance of these bioactive compounds in a more complex metabolic environment.

## Conflicts of Interest

The authors declare no conflicts of interest.

## Data Availability

The data used to support the findings of this study are included within the article.
